# Electroacupuncture at ST25 Inhibits Cisapride-Induced Gastric Motility in an Intensity-Dependent Manner

**DOI:** 10.1155/2016/3457025

**Published:** 2016-02-22

**Authors:** Tingting Pang, Chunxia Lu, Kaiyue Wang, Chao Liang, Zhi Yu, Bing Zhu, Bin Xu

**Affiliations:** ^1^Key Laboratory of Integrated Acupuncture and Drugs, Nanjing University of Chinese Medicine, 138 Xianlin Road, Qixia District, Nanjing, Jiangsu 210023, China; ^2^Institute of Acupuncture and Moxibustion, China Academy of Chinese Medical Sciences, Beijing, China

## Abstract

*Background.* Previous studies have demonstrated the efficacy of frequency-specific EAS. However, evaluation of intensity-response effects is challenging and has yet to be addressed.* Aims.* Using cisapride to promote gastric emptying, we measured the intensity-response relationship of EA at ST25 on gastric motility.* Methods.* We determined the effects of EA at ST25 using intensities (0.5, 1, 3, 5, 7, and 9 mA) on gastric motility in rats injected with cisapride (0.2, 0.02, and 0.002 mg/kg).* Results.* Utilizing three concentrations of cisapride yielded significantly differing levels of gastric motility. Furthermore, log IC_50_ values for EAS were different within each group. Given the same EA intensity, cisapride antagonism decreased progressively in each group as a function of drug concentration. The relative amount of cisapride antagonized by EAS did not change in a linear fashion. Finally, EAS at different intensities within the three groups induced a similar pattern of cisapride antagonism.* Conclusions.* The ability of EAS to elicit a decrease in cisapride-induced gastric motility pressure was demonstrated in this study. The study encompasses construct validity to mirror individualized treatment being based on patients' subjective feelings, not on a set fixed EA intensity. Clinically utilizing EAS at the smallest intensity can achieve the desired therapeutic effect.

## 1. Introduction

Acupuncture is a branch of traditional Chinese medicine that has been used extensively and safely for the treatment of various diseases in China for over 5000 years. In comparison with conventional acupuncture, electroacupuncture (EA) treatment is capable of increasing the stimulation frequency and intensity in a controlled and quantifiable manner. Several studies have demonstrated the efficacy of electrical stimulation in EA. Some studies have shown that different neuropeptides in the central nervous system can be triggered by EA of different frequencies and that different frequencies of stimulation can induce different peripheral reactions [1, 2].

One study found that, in comparison with 1 mA electrical stimulation, a 3 mA electrical stimulation intensity was not associated with a greater therapeutic effect [3]. Su et al. studied the electrical intensities of 0.5, 1, 3, 5, 7, and 9 mA in EA in activating C fibers [4]. They found that 2.1–2.3 mA was the half-maximal facilitation intensity of EAS at acupuncture point ST36 and that 2.8 mA was the half-maximal inhibitory intensity (IC_50_) of EAS at acupuncture point CV12 [4]. However, reports focusing on the efficacy of the intensity of electrical stimulation (as well as the relationship between different intensities of EA and different excited states) are lacking.

5-Hydroxytryptamine (5-HT), or serotonin, is an important neurotransmitter in the brain and gut. The gastrointestinal (GI) tract contains 95% of the 5-HT in the body and is involved in secretion and motility within the GI tract [5]. 5-HT receptors have been classified into five subtypes (1, 2, 3, 4, and 7) [6]. 5-HT4 receptors are expressed on the ends of cholinergic nerves in the enteric nervous system, and activation of 5-HT4 receptors can stimulate GI propulsive motility by increasing the release of acetylcholine from motor neurons [7, 8]. 5-HT4 receptor agonists are used to promote gastric emptying. The nonselective 5-HT4 receptor agonist cisapride was developed for the treatment of gastroesophageal reflux disease and dyspepsia [9, 10]. Here, we used cisapride as the ideal drug to measure the effective dose of EAS. We also investigated the relationship between the effect and intensity of EAS at acupuncture point Tianshu (ST25) upon gastric motility.

## 2. Materials and Methods

### 2.1. Ethical Approval of the Study Protocol

The study protocol was approved by the Scientific Investigation Board of Nanjing University of Traditional Chinese Medicine (Nanjing, China). All experimental procedures were undertaken in accordance with those detailed in the* Guide for the Care and Use of Laboratory Animals* (National Institutes of Health, Bethesda, MD, USA). We strived to minimize rat suffering and to reduce the number of rats used in the study.

### 2.2. Animals

All experiments utilized adult male Sprague-Dawley rats (180–230 g; Model Animal Research Center of Nanjing Medical University). Rats were allowed* ad libitum* access to food and water. The room in which the rats were housed was strictly controlled: temperature (22°C), illumination (12-h light-dark cycle), and humidity (40–60%). Rats were allowed to acclimate to their housing environment for 1 week before experimentation.

### 2.3. Drugs

Urethane was purchased from Sigma-Aldrich (Saint Louis, MO, USA). Cisapride monohydrate (Sigma-Aldrich) was dissolved in vehicle (10% dimethyl sulfoxide; Sigma-Aldrich). Urethane (20%, 0.8 g/kg) and cisapride (0.2, 0.02, and 0.002 mg/kg) [11–13] were used in separate cohorts of rats.

### 2.4. Experimental Procedure

**Figure 1:**
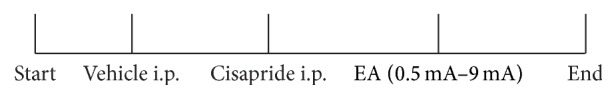
Timeline of the experimental procedure.

After 12 hours of fasting overnight and* ad libitum* access to water, rats were anesthetized with urethane (0.8 g/kg, i.v.). The trachea was cannulated to ensure that the respiratory tract was not obstructed. A small incision was made 1 cm from the xiphoid in the abdomen. A small balloon composed of flexible rubber was inserted into the gastropyloric region via the incision in the duodenum. A transducer (YP201, Chengdu Instrument Factory, Chengdu, China) was used to measure balloon pressure. A physiologic signal-acquisition system (RM6240; Chengdu Instrument Factory) collected the signal for further analyses. Baseline pressure was maintained at 0.1–0.5 kPa. During the experiment, an electric heating board maintained the rat's temperature at 37 ± 0.5°C. Escalating intensities of EA (0.5, 1, 3, 5, 7, and 9 mA) were applied at Tianshu (ST25), with each EA stimulation intensity level being delivered for 1 minute. Only when gastric motility recovered to baseline levels could stimuli be applied. In the cisapride treatment group, vehicle was delivered for ~30 minutes prior to cisapride. Different concentrations of cisapride were injected, so the times for the steady state of gastric motility to be reached were different: 0.1 mg/mL of cisapride, ≈40–60 minutes; 0.01 mg/mL of cisapride, ≈60–90 minutes; 0.001 mg/mL of cisapride, ≈120–150 minutes. The experimental procedure is represented in Figure 1.

### 2.5. EAS

During electroacupuncture stimulation (EAS) trials, a pair of needles (diameter: 0.18 mm) were used at Tianshu (ST25, unilateral). ST25 is located bilaterally 5 mm lateral to the intersection between the upper two-thirds and lower one-third in the line between the xiphoid process and upper border of the pubic symphysis [14]. EA frequency was set at 2/15 Hz with continuous stimulation for 1 minute at different intensities (0.5 mA to 9 mA). Needles were connected to an EA apparatus (HANS-200; Nanjing Jisheng Medical Technology, Nanjing, China).

### 2.6. Assessment

Gastric motility during EA was compared to that before EA was initiated (i.e., pre-EA). If the gastric motility fold change rate was <100%, the response was considered to be “inhibited.” The fold change rate was fit with (1); the unit of EA was applied here to evaluate the efficiency of EA. The fold change of gastric motility induced by unit of EA intensity was fit with (2). Consider

(1) Y=preEAdurEA×100%,

(2) Y=preEA−durEApreEA×intensity×100%.

### 2.7. Statistical Analyses

All results are displayed as mean ± standard error. Values recorded before and after administration were analyzed using a paired-sample *t*-test. The results obtained in the three groups were compared using one-way ANOVA. All *p* values <0.05 were considered statistically significant. Data were analyzed using the software program SPSS version 18.0 (IBM, Armonk, NY, USA). The data curve of different intensities was fitted with (3) where *X* is the log of intensity, *Y* is the response (rate of fold change as *X* increases), “Top” and “Bottom” are plateaus in the same units as *Y*, and log IC_50_ has the same log units as *X*:

(3) $$\begin{array}{c} Y=\text{Bottom}+\frac{\left(\text{Top}-\text{Bottom}\right)}{1+10^{X-\text{LogIC50}}}. \end{array}$$

## 3. Results

### 3.1. Cisapride Concentration and Gastric Motility

In anesthetized rats, gastric baseline pressure was maintained at 0.1–0.4 kPa by increasing balloon volume to 0.1–0.2 mL with warm water. When gastric contractions stabilized, we investigated the effect of EAS at ST25 upon gastric motility.

First, cisapride (0.2, 0.02, or 0.002 mg/kg) was injected intraperitoneally in three separate cohorts of rats (*n* = 6 per group). After 45–60 minutes, significantly enhanced gastric motility was observed in rats treated with 0.2 mg/kg cisapride (henceforth termed “Group 1”); gastric pressure was 0.35–0.53 kPa. In rats treated with 0.02 or 0.002 mg/kg cisapride (“Group 2” and “Group 3,” resp.), gastric motility increased after 60–90 minutes and 120–150 minutes, respectively, with gastric pressures of 0.29–0.76 kPa and 0.24–0.42 kPa, respectively.

**Figure 2:**
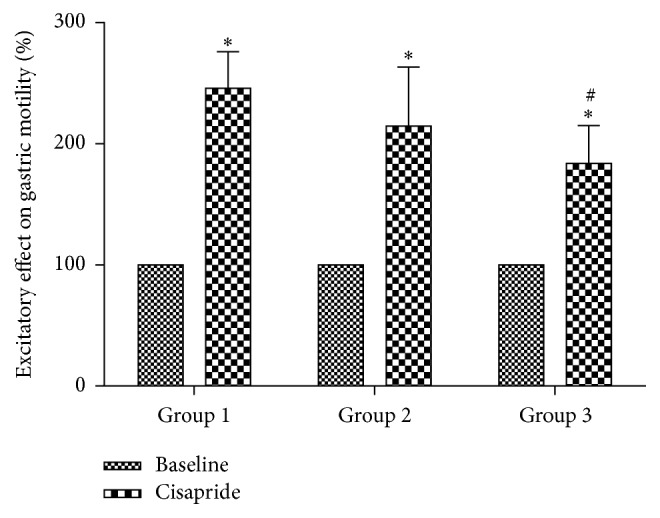
Comparisons of gastric motility after injection of different concentrations of cisapride. ^*∗*^ *p* < 0.01 versus baseline (preadministration of cisapride); ^#^ *p* < 0.01, comparison between groups. A significant difference in gastric motility was observed between Group 1 and Group 3 (Group 1: 0.2 mg/kg cisapride, Group 2: 0.02 mg/kg cisapride, and Group 3: 0.002 mg/kg cisapride).

Therefore, different levels of gastric motility were observed before and after injection with three concentrations of cisapride, and these differences were significantly different from one another (*p* < 0.01) (Figure 2). Moreover, gastric motilities were different between the three groups, but only the difference between Group 1 and Group 3 was statistically significant (*p* < 0.01). In terms of trend, as the concentration of cisapride injected increased, the gastric pressure also increased.

### 3.2. EA Intensity and Gastric Motility

After the gastric pressure reached a peak, different EA intensities (0.5, 1, 3, 5, 7, and 9 mA) were applied to the three rodent cohorts in ascending order for one-minute duration each at ST25.

**Figure 3:**
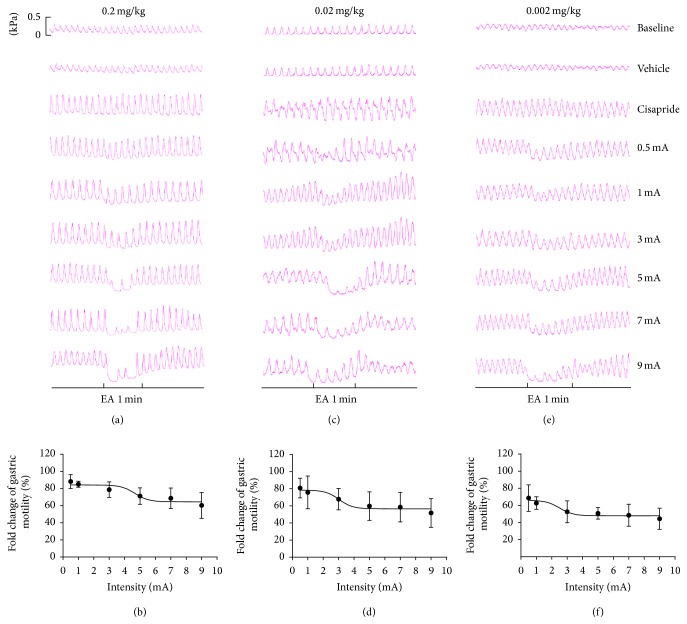
Gastric motility in response to EAS at ST25 with different intensities in the three cisapride treatment groups. (a) Representative traces of alterations in gastric contractions induced by different intensities of EA at ST25 in Group 1. (b) Intensity-response relationship between EAS intensity (mA) and the effect on cisapride-induced gastric motility (0.2 mg/kg). (c) Representative traces of alterations in gastric contractions induced by different intensities of EA at ST25 in Group 2. (d) Intensity-response relationship between EAS intensity (mA) and the effect on cisapride-induced gastric motility (0.02 mg/kg). (e) Representative traces of alterations of gastric contractions induced by different intensities of EA at ST25 in Group 3. (f) Intensity-response relationship between EAS intensity (mA) and the effect on cisapride-induced gastric motility (0.002 mg/kg).

In Group 1, the fold changes in gastric motility for EA intensities of 0.5, 1, 3, 5, 7, and 9 mA were 88.22 ± 0.04%, 85.06 ± 3.51%, 78.74 ± 9.11%, 71.25 ± 9.65%, 68.68 ± 11.95%, and 60.38 ± 15.07%, respectively. The log IC_50_ value of stimulation upon administration of 0.2 mg/kg cisapride into rats was 4.67 ± 0.49 mA (Figures 3(a) and 3(b)). When EAS < 7 mA, the response to EAS increased with increasing of EA intensity, but at 7 mA it reached a plateau.

In Group 2, the fold changes in gastric motility for EA intensities of 0.5, 1, 3, 5, 7, and 9 mA were 80.83 ± 11.43%, 75.74 ± 19.09%, 67.86 ± 12.52%, 59.71 ± 16.01%, 58.56 ± 17.29%, and 51.74 ± 16.82%, respectively. The effect of EAS at ST25 on gastric motility after administration of 0.02 mg/kg cisapride was similar to the result for Group 1, but the log IC_50_ value was reduced to 3.03 ± 0.56 mA (Figures 3(c) and 3(d)). When EA intensity was <5 mA, response to EAS increased as intensity increased. When EA intensity was >5 mA, the response increased slowly and reached a plateau; that is, the inhibitory effect did not increase with increasing intensity.

In Group 3, the fold changes in gastric motility for EA intensities of 0.5, 1, 3, 5, 7, and 9 mA were 68.57 ± 15.49%, 62.73 ± 7.36%, 52.54 ± 12.76%, 50.67 ± 6.72%, 48.48 ± 11.36%, and 44.38 ± 12.38%, respectively. The log IC_50_ value for EAS was 2.50 ± 0.69 mA (Figures 3(e) and 3(f)). The response to EAS increased with increasing EA intensity until a plateau was reached at 4 mA.

### 3.3. EA and Effective Dose

In Group 1 (injected with 0.2 mg/kg cisapride), for gastric motility to increase by 10%, 3.39 ± 0.90 *μ*g of cisapride was needed. In Groups 2 and 3, for gastric motility to increase by 10%, 0.33 ± 0.07 *μ*g and 0.05 ± 0.01 *μ*g of cisapride were needed, respectively. At identical EA intensity, the cisapride concentration required for gastric motility to increase by 10% decreased progressively from Group 1 to Group 3. The cisapride concentration changed tenfold between Group 3 and Group 1, but the dose of cisapride antagonized by EAS did not change in a linear fashion (Tables 1–3).

### 3.4. Decrease in Gastric Motility Pressure between Groups

**Figure 4:**
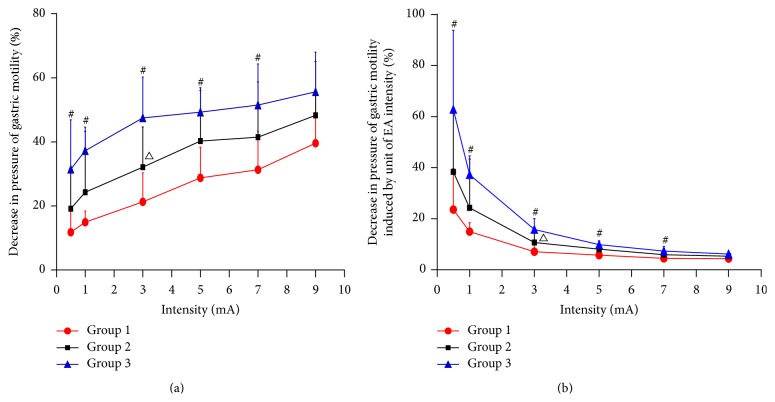
(a) Decrease in gastric motility pressure produced by EA intensity. (b) Decrease in gastric motility pressure produced by one unit of EA intensity. ^#^ *p* < 0.05, Group 1 versus Group 3; Group 1 versus Group 2; ^∆^ *p* < 0.05, Group 2 versus Group 3.

Figure 4(a) details the decrease in gastric motility pressure elicited by different EA intensities, and the three lines representing the three groups do not intersect. This finding suggests that the trend of EAS in the three groups at different intensities antagonizes cisapride in a similar fashion. The only significant difference in decrease of gastric motility pressure was between Groups 1 and 3 (*p* < 0.05), except at 9 mA.  Figure 4(b) shows that, in each group, the decrease in gastric motility pressure produced by one unit of EA intensity diminished as EA intensity increased. Additionally, at >3 mA, the decrease in gastric motility pressure produced by one unit of EA intensity in each group changed only negligibly. Between Groups 2 and 3, a significant difference was observed at 1 and 3 mA intensity. Additionally, at 0.5, 1, 3, and 5 mA, there was significant difference between Groups 1 and 2 (*p* < 0.05).

## 4. Discussion

Western medicine typically uses fixed dose or fixed-dose combinations to treat individual diseases, for example, for adults, in most cases, the western medicine is usually taken twice or three times a day, one pill or more at a time, but only for children, the dose should be reduced. It would be once or twice a day, 1/2,1/4, or less than one pill at a time or another less dose. The dose is always fixed. However, in Chinese medicine, the dose is based on the symptoms and physical signs, and, furthermore, the same disease with different syndromes could conform to different Chinese herbs with different dosages. That is taken for treatment based on differentiation. There should be a great difference between western medicine and Chinese medicine. In Chinese medicine, acupuncture is an important and commonly used treatment modality. It is becoming increasingly popular both within and beyond China, and the use of EAS has gained widespread acceptance as a viable treatment option. During treatment, the medical practitioner gauges the therapeutic effect upon the individual patient's reported subjective feelings and treatment tolerance. An important remaining question is whether the effective intensity of EAS is also fixed. As previously noted, there has been a great deal of published research concerning the optimal frequency of EA [1, 2]. There is also some literature regarding EA intensity effects [15]. However, to date, there is very little known about the intensity-response effect of EA, in either clinical trials or preclinical experiments. We aimed to determine whether the effective intensity of EAS is fixed in EA, analogous to the fixed doses of drugs commonly prescribed in Western medicine. Therefore, we designed this study to specifically investigate the intensity-response effect of EA.

Gastric motility was inhibited by acupuncture-like stimulation applied to the abdomen and lower chest in a study by Sato and colleagues [16]. Several researchers have confirmed that EA at the acupoint ST25 significantly inhibits gastric motility via the sympathetic nervous system [17–23]. The nonselective 5-HT4 receptor agonist cisapride reliably promotes gastric emptying. Hence, in the present study, we investigated the intensity-response relationship using ST25 and cisapride. We found that different levels of gastric motility could be triggered when three concentrations of cisapride were utilized and that these motility levels were significantly different from one another. Cisapride is an ideal pharmacological agent to model a singular gastrointestinal disease encompassing varying degrees of severity. Between each cisapride concentration used, the log IC_50_ values after EAS were significantly different. That is, at different levels of the same disease, the intensity of EAS was not fixed. Therefore, our pharmacological model fully encompasses construct validity to mirror individualized treatment being based on patients' feelings, not on a fixed EA intensity.

Given the same EA intensity in all three groups, cisapride antagonism decreased progressively as a function of drug concentration (Tables 1–3). Also, in one group, as EA intensity increased, the relative cisapride dose antagonized by EAS increased. The cisapride concentration changed tenfold from Group 3 to Group 1; however, the relative amount of cisapride antagonized by EAS did not change in a linear fashion. For the first time, this study suggests that the intensity-response effects of EA can change along with changes in the state of the periphery.

We used the EA intensity unit to investigate the intensity-response effect of EA. Figures 4(a)-4(b) revealed that, given an identical EA intensity between the three groups, different cisapride concentrations can elicit different responses. Furthermore, EAS at different intensities within the three groups caused a similar pattern of cisapride antagonism.  Figure 4(b) shows that, in each group, the decrease in gastric motility pressure produced by one unit of EA intensity decreased as EA intensity increased. Therefore, the same intensity of EAS in a singular disease with varying degrees of severity could elicit different responses and could be modified along with changes in the body. The effect caused per unit of EA intensity within the intensity range of 0.5 mA to 3 mA was greater than that within the intensity range of 5 mA to 9 mA. This suggests that similarly, in the clinic, EAS beginning at a small intensity can reliably evoke the desired therapeutic effect in the patient.

## 5. Conclusions

The present study demonstrated a significant decrease in gastric motility pressure induced by EAS. The effects caused by identical EAS intensities could be modified based on bodily homeostatic alterations. Therefore, we conclude that individualized EA treatment should be based upon the patients' subjective feelings, and not on a fixed intensity of EA. Additionally, in the clinic, EAS initially utilizing a small EA intensity can always achieve the desired therapeutic effect.

## Figures and Tables

**Table 1:** Gastric motility responses to different EA intensities at ST25 using 0.2 mg/kg cisapride.

	Cisapride efficiency	0.5 mA	1 mA	3 mA	5 mA	7 mA	9 mA
Rate of change	10%	11.78 ± 8.04	14.94 ± 3.51	21.26 ± 9.11	28.75 ± 9.65	31.32 ± 11.95	39.62 ± 15.06
Cisapride dosage	3.39 ± 0.90	3.64 ± 2.83	4.64 ± 1.23	6.83 ± 3.20	9.37 ± 3.39	10.43 ± 4.20	13.22 ± 5.29

**Table 2:** Gastric motility responses to different EA intensities at ST25 using 0.02 mg/kg cisapride.

	Cisapride efficiency	0.5 mA	1 mA	3 mA	5 mA	7 mA	9 mA
Rate of change	10%	19.17 ± 11.43	24.26 ± 19.09	32.14 ± 12.52	40.29 ± 16.61	41.44 ± 17.29	48.26 ± 16.82
Cisapride dosage	0.33 ± 0.07	0.63 ± 0.37	0.83 ± 0.60	1.05 ± 0.41	1.32 ± 0.54	1.36 ± 0.57	1.58 ± 0.55

**Table 3:** Gastric motility responses to different EA intensities at ST25 using 0.002 mg/kg cisapride.

	Cisapride efficiency	0.5 mA	1 mA	3 mA	5 mA	7 mA	9 mA
Rate of change	10%	31.43 ± 15.49	37.27 ± 7.36	47.46 ± 12.76	49.32 ± 6.72	51.52 ± 12.83	56.62 ± 12.38
Cisapride dosage	0.05 ± 0.01	0.21 ± 0.08	0.31 ± 0.04	0.64 ± 0.07	0.93 ± 0.03	1.23 ± 0.07	1.53 ± 0.06
